# Breast Cancer Detection and Prevention Using Machine Learning

**DOI:** 10.3390/diagnostics13193113

**Published:** 2023-10-02

**Authors:** Arslan Khalid, Arif Mehmood, Amerah Alabrah, Bader Fahad Alkhamees, Farhan Amin, Hussain AlSalman, Gyu Sang Choi

**Affiliations:** 1 Faculty of Computing, Islamia University of Bahawalpur, Bahawalpur 63100, Punjab, Pakistan; arslankhalid436@gmail.com (A.K.); arifnhmp@gmail.com (A.M.); 2 Department of Information Systems, College of Computer and Information Science, King Saud University, Riyadh 11543, Saudi Arabia; balkhamees@ksu.edu.sa; 3 Department of Information and Communication Engineering, Yeungnam University, Gyeongsan 38541, Republic of Korea; castchoi@yu.ac.kr; 4 Department of Computer Science, College of Computer and Information Sciences, King Saud University, Riyadh 11543, Saudi Arabia; halsalman@ksu.edu.sa

**Keywords:** breast cancer, healthcare, machine learning

## Abstract

Breast cancer is a common cause of female mortality in developing countries. Early detection and treatment are crucial for successful outcomes. Breast cancer develops from breast cells and is considered a leading cause of death in women. This disease is classified into two subtypes: invasive ductal carcinoma (IDC) and ductal carcinoma in situ (DCIS). The advancements in artificial intelligence (AI) and machine learning (ML) techniques have made it possible to develop more accurate and reliable models for diagnosing and treating this disease. From the literature, it is evident that the incorporation of MRI and convolutional neural networks (CNNs) is helpful in breast cancer detection and prevention. In addition, the detection strategies have shown promise in identifying cancerous cells. The CNN Improvements for Breast Cancer Classification (CNNI-BCC) model helps doctors spot breast cancer using a trained deep learning neural network system to categorize breast cancer subtypes. However, they require significant computing power for imaging methods and preprocessing. Therefore, in this research, we proposed an efficient deep learning model that is capable of recognizing breast cancer in computerized mammograms of varying densities. Our research relied on three distinct modules for feature selection: the removal of low-variance features, univariate feature selection, and recursive feature elimination. The craniocaudally and medial-lateral views of mammograms are incorporated. We tested it with a large dataset of 3002 merged pictures gathered from 1501 individuals who had digital mammography performed between February 2007 and May 2015. In this paper, we applied six different categorization models for the diagnosis of breast cancer, including the random forest (RF), decision tree (DT), k-nearest neighbors (KNN), logistic regression (LR), support vector classifier (SVC), and linear support vector classifier (linear SVC). The simulation results prove that our proposed model is highly efficient, as it requires less computational power and is highly accurate.

## 1. Introduction

Cancer is a worldwide epidemic that affects individuals of all ages and backgrounds. There are many types of cancer, however, breast cancer is one of the most common cancers in women. Due to this challenge, researchers should pay special attention to cancer detection and prognosis. Predicting and diagnosing cancer at an early stage is an area where machine-learning approaches may have a significant impact. Breast cancer develops from breast cells and is a frequent malignancy in females worldwide. Breast cancer is second only to lung cancer as a leading cause of death in women [1]. The risk of breast cancer can be due to the following causes: age is a major factor in the development of breast cancer. Breast cancer is more prevalent in females, but males are not immune to the disease. Having a mother, sister, or daughter who has been diagnosed with breast cancer increases an individual’s risk. Breast cancer risk increases with the presence of certain gene mutations, such as those in the BRCA1 and BRCA2 genes. The use of HRT (hormone replacement therapy) for an extended period may raise the risk. Premature menstruation, delayed menopause, and a lack of children or delayed puberty are all factors that might affect a woman’s risk. The symptoms of breast cancer are the following: isolated swelling of the breast or armpit; alterations in breast size, form, or appearance; breast or nipple discomfort which cannot be explained medically; nipple discharge other than breast milk; and discoloration or dimples in the breast skin. The microscopic appearance of breast cancer cells is used to classify the disease into several subtypes. Ductal Carcinoma In Situ (DCIS) is a non-invasive or pre-invasive breast cancer that originates in milk ducts and does not invade nearby tissues. It can sometimes progress to invasive breast cancer if left untreated. Invasive ductal carcinoma (IDC) is the most common type, accounting for 80% of cases, and invades nearby breast tissues. IDC can be classified based on hormone receptor status and HER2 status. Invasive lobular carcinoma (ILC) originates in the milk-producing glands and accounts for 10–15% of breast cancer cases. It is less common than IDC and is more difficult to detect with mammography. Triple-negative breast cancer (TNBC) is a subtype of breast cancer that lacks hormone receptors and the HER2 protein. It is more aggressive and less responsive to hormonal and HER2-targeted therapies. TNBC is often treated with chemotherapy. HER2-positive breast cancer is a rare and aggressive form of breast cancer caused by overexpression of the human epidermal growth factor receptor 2 (HER2) protein. It can be invasive or non-invasive, and it can be treated with targeted therapies such as Herceptin. Inflammatory breast cancer (IBC) is a rare and aggressive form characterized by redness, warmth, and swelling of the breast. It requires immediate and aggressive treatment. Paget ’s disease of the Nipple is a rare and aggressive form that starts in the milk ducts and spreads to the skin of the nipple and areola. Metastatic breast cancer, also known as stage IV, is a non-curable disease that can be any subtype of breast cancer (IDC, ILC, or HER2-positive) and is generally not curable. Treatments aim to control and manage the disease. Invasive ductal carcinoma (IDC) and ductal carcinoma in situ (DCIS) are the two most common forms of breast cancer, with DCIS developing more slowly and usually without affecting patients’ everyday lives. DCIS accounts for a very modest number of breast cancer instances (20–53%), while IDC is far more hazardous since it spreads across the breast. About 80% of people diagnosed with breast cancer fall into this group [2]. The majority of breast cancers are invasive, indicating that the illness has spread to other organs or tissues beyond the breast. The two most common types of invasive breast cancer are invasive ductal carcinoma (IDC) and invasive lobular carcinoma (ILC). Invasive ductal carcinoma is a kind of breast cancer that develops in the milk ducts and then metastasizes to other parts of the breast. Invasive lymphoma is more difficult to detect using standard screening procedures. Initially developing in the lobules that secrete milk, inflamed lobular carcinoma (ILC) may spread to other parts of the breast [3]. Only 1–4% of women with a breast cancer diagnosis will go on to develop invasive ductal carcinoma in situ, a type of breast Paget disease. It is an uncommon tumor that develops in the skin surrounding the breasts and produces Paget cells. Due to its rapid growth rate, breast angiosarcoma is often diagnosed after it has already spread to other parts of the body. The phyllodes seldom become malignant growths, and when they do, they mostly affect middle-aged women. The other types of invasive breast cancer include adenocarcinoma, adenosquamous carcinoma, medullary carcinoma, mucinous carcinoma, papillary carcinoma, and tubular carcinoma, each of which is very uncommon [4]. Cancer cells in a noninvasive breast tumor stay localized inside the affected breast region rather than spreading to nearby lobules or ducts. In the case of breast cancer, the disease has not spread beyond the affected area. There are two types of in situ cancer: ductal carcinoma in situ (DCIS) and lobular carcinoma in situ (LCIS) [5]. Cancer of the milk duct, or ductal carcinoma in situ (DCIS), occurs when a mass forms within the duct that carries milk from the lobules or glands to the nipple.

Recent advances in diagnostic and treatment methods have led to a positive prognosis for those who have been treated for DCIS, even if there is no proof that cancer has spread to other organs [6]. However, LCIS may promote the growth of cells that resemble cancerous ones, so it is important to treat it as if it were. Most cases of LCIS remain localized; however, because of the increased risk of invasive breast cancer, doctors may opt to monitor their patients anyway. Early detection of cancer is key to the successful treatment of breast cancer. Therefore, the availability of appropriate screening technologies is crucial for spotting the first signs of breast cancer. Screening for this condition may be performed using a variety of imaging modalities, the most common of which are mammography, ultrasonography, and thermography. Mammography is an important tool for detecting breast cancer in its earliest stages. Since mammography is ineffective for women with dense breast tissue, diagnostic ultrasound is often used instead. Radiation from radiography and thermography may be more accurate than ultrasonography for detecting tiny malignant tumors due to these factors. Mammography is a crucial tool in the fight against breast cancer, and thanks to advancements in artificial intelligence, it can now automatically identify illnesses in medical photos. Early-stage breast cancer detection faces several limitations and challenges. Breast cancer screening methods, such as mammography, face several challenges, including sensitivity, specificity, breast density, overdiagnosis, overtreatment, screening age and frequency, access and equity, patient compliance, cost, and resource constraints, a false sense of security, risk prediction, genetic and molecular factors, variability in screening interpretation, and invasive follow-up tests. Mammography may miss early-stage tumors, particularly in women with dense breast tissue, resulting in false negatives and unnecessary follow-up tests. Dense breast tissue can obscure small tumors on mammograms, reducing sensitivity and requiring additional imaging tests. Overdiagnosis and overtreatment can occur when early-stage breast cancers are slow-growing and non-aggressive. Screening age and frequency may vary by country and organization, leading to confusion among patients and healthcare providers. Access to breast cancer screening can be limited by socioeconomic factors, geographic location, and healthcare disparities, resulting in missed opportunities for early detection. Patient compliance, cost and resource constraints, and genetic and molecular factors also contribute to the challenges faced in breast cancer screening. Patients anxiously anticipating biopsy findings indicating benignity may be subjected to unnecessary follow-up workups and biopsies if the diagnostic yield is only moderately specific. Over the last several decades, methods have been included in the normal clinical evaluation of breast MRI tests that examine various MRI [7] sequences alongside DCE-MRI pictures to overcome this constraint and analyze more functional data. T2-weighted (T2w) MRI is an often-used supplementary sequence in this method, which is known as multi-parametric magnetic resonance imaging (mpMRI). Differentiating benign from malignant lesions may be aided by including T2W sequence interpretation, according to studies. As an example, fibroadenomas, a benign lesion that may demonstrate comparable contrast agent enhancement to that of malignant lesions on T1-weighted DCE-MRI, often have higher signal intensity on T2w images compared with malignant lesions [8]. Instruments have been produced to make and enhance image processing because of the inherent challenges connected with a picture, such as low contrast, noise, and underappreciation by the human eye. Convolutional neural networks (CNNs) [9], a subfield of machine learning, and artificial intelligence (AI) are some of the healthcare industry’s hottest new trends. Artificial intelligence (AI) [10] and machine learning (ML) may be found in the field of study that focuses on developing better technological systems to handle complicated tasks with less reliance on human intellect [11].

The purpose of this research is to create an efficient deep learning-based model. The proposed model is capable of recognizing breast cancer in computerized mammograms of varying densities and then comparing the achieved results using state-of-the-art models. We proposed an efficient deep learning model that is capable of recognizing breast cancer in computerized mammograms of varying densities. Our research relied on three distinct modules for feature selection: the removal of low-variance features, univariate feature selection, and recursive feature elimination. The craniocaudally and medial-lateral views of mammograms are incorporated. We suggested craniocaudally and medial-lateral views of mammograms in our proposed model. This resulted in a total of 3002 merged pictures from 1501 individuals who had digital mammography performed between February 2007 and May 2015. It has been observed that breast MRI is a very sensitive imaging technique for detecting and characterizing breast cancer. We obtained excellent sensitivity and varied specificity for breast cancer. The diagnosis was obtained using dynamic contrast-enhanced (DCE) MRI. This provides morphological and functional lesion information.

The Table 1 gives a concise overview of different diagnostic techniques along with both benefits and drawbacks. This serves as a fast reference for readers to comprehend the state of breast cancer diagnosis today and the need for an enhanced diagnostic strategy.

### 1.1. Motivation

Breast cancer diagnosis by machine learning has been motivated by the hope that it will lead to better patient outcomes, lessen the disease’s worldwide effect, and aid in the development of cutting-edge healthcare technology and research.

### 1.2. Benefits of This Research

The benefits of this research are as follows; it helps in improvement in early diagnosis and individualized therapy. In addition, it has the potential to revolutionize breast cancer management and save lives by influencing areas such as research, cost-effectiveness, and worldwide accessibility to healthcare services. It helps in reducing healthcare costs, and a more beneficial influence on worldwide breast cancer management is all possible because of the abilities of machine learning in breast cancer diagnosis.

The rest of the paper is organized as follows. In Section 2 we discuss the previous research in the related work section. In Section 3 we presented our proposed methodology. In Section 4 we discuss the achieved experimental results. Section 5 concludes the conclusion of this study.

## 2. Literature Review

In recent years, several studies have used ML (machine learning) techniques in healthcare domains to detect BC. Since the algorithms provide satisfactory results, other scientists have used them to address challenging issues [12]. A CNN algorithm was employed to predict and diagnose invasive ductal carcinoma in breast cancer images, and it achieved an accuracy of about 88% [13,14]. In addition, it is often used in the medical field for forecasting and diagnosing anomalous occurrences to obtain a deeper understanding of incurable disorders such as cancer [15]. Numerous studies have focused on breast cancer detection strategies that use imaging and genetics. Furthermore, to our knowledge, no studies have been conducted that use both of these approaches together. In [16], the authors summarized the several techniques used for histological image analysis (HIA) in breast cancer diagnosis. Different types of convolutional neural networks (CNN) serve as the foundation for these techniques [17]. Based on the kind of dataset they used, the writers classified their work accordingly. They organized everything in reverse chronological order, with the most recent occurrence at the beginning. This study’s results suggest that ANNs were first put to use in the area of HIA sometime in the middle of 2012. The most common types of algorithms used were ANNs and PNNs [18].

However, morphological and textural characteristics were heavily used in feature extraction. It is clear that using deep convolutional neural networks to detect and diagnose breast cancer at an early stage improves outcomes for patients undergoing treatment. The process of creating NCD predictions included the use of several different algorithms. In [19], the authors investigated and evaluated several categorization strategies for their effectiveness. The classification algorithms were tested on eight separate NCD datasets using a 10-fold cross-validation strategy. The area under the curve is used to analyze these results for precision. The authors state that the NCD datasets have irrelevant features and noisy data. The resiliency of KNN, SVM, and NN in the face of this noise is impressive. They also suggested various preprocessing processes that would raise the rate of accuracy and remove the problem of irrelevant attributes. Several human health disorders have been presented as candidates for which natural inspiration computing (NIC) approaches might be useful in the diagnostic process. The authors of [20] proposed five NIC diagnostic algorithms based on insects and addressed their potential use in diagnosing diabetes and cancer. Breast, lung, prostate, and ovarian tumors were all successfully recognized, as claimed by the authors. A breast cancer diagnosis is improved by integrating directed ABC with neural networks. The authors also developed a very effective technique for identifying diabetes and leukemia. Incorporating NICs with conventional classification techniques, they reasoned, yields more reliable and encouraging results. They stressed the need for further research into diabetes and illness detection at different stages. In [21], the authors reported data suggesting NNs may be used to classify cancer diagnoses, especially in the early stages of the illness. Their findings show that a variety of NNs have shown promise in identifying cancerous cells. However, a significant amount of computing power is required for the imaging method’s preprocessing of the pictures. In the following, we explore how CNNs and AI can minimize challenges.

CNNs and AI can improve medical image quality by enhancing low-contrast features, reducing noise, removing artifacts, and optimizing image registration. They can also assist in image, segmentation, and ROI detection, enabling precise analysis and diagnosis of anatomical structures or lesions. AI algorithms can adjust image contrast, brightness, and intensity levels and apply contrast-limited adaptive histogram equalization (CLAHE) techniques to improve image quality. Additionally, CNNs can recognize and remove common imaging artifacts, ensuring accurate interpretation. AI algorithms optimize image alignment, while segmentation and ROI detection enable precise analysis and diagnosis of specific areas. Finally, CNNs can be used for super-resolution imaging, enhancing image resolution and quality beyond the original acquisition. AI-driven super-resolution techniques use deep learning models to generate high-resolution images from low-resolution inputs, providing enhanced detail and diagnostic information.

Practical techniques in the field of smart health include computational intelligence methods such as fuzzy systems, artificial neural networks, and swarm intelligence, or evolutionary computing methods including genetic algorithms, classifiers, and support vector machines (Al-Antari, Al-Masni et al., 2018) [22]. The suggested CNN Improvements for Breast Cancer Classification (CNNI-BCC) model helps doctors spot breast cancer, as shown in research in Khan, Khan et al., 2020) [21]. The suggested method uses a trained deep learning neural network system to categorize breast cancer subtypes. According to data from 221 actual patients, the findings have an accuracy of 90.50 percent. Without needing any human intervention, this model can classify and identify breast cancer lesions. Evaluating this model shows that it can examine the situation of impacted patients throughout the detection phase, showing that it is an improvement over earlier techniques (Tanabe, Ikeda et al., 2020) [23].

Sivapriya, Kumar, et al., 2019 [24], compared SVM, logistic regression, naive Bayes, and random forest to determine their parallels and distinctions. Wisconsin’s breast cancer dataset is used for comparative purposes (Abunasser, AL-Hiealy et al., 2022) [25]. The results of the evaluations showed that the random forest algorithm achieved the highest level of accuracy (99.76%) with the least amount of error. The Anaconda Data Science Platforms were used to run all the experiments in a reproducible environment [26]. The authors (Allugunti 2022) proposed an approach for breast cancer that classifies the disease into its various subgroups. Features are chosen using data from the Wisconsin Diagnosis and Analysis and Prognostic Breast Cancer databases (Gonzalez-Angulo, Morales-Vasquez et al., 2007) [27]. The different types of breast cancer are then categorized using a neural network technique, with special emphasis on the multilayer perceptron (MLP) and the back-propagation neural RBF. The nine characteristics in this dataset stand for the neural network’s input layer. The neural network will classify the input information into two types of cancer (benign and malignant). Using the RBF neural network, the method developed and evaluated on the database achieved a 97% repeatability of classification. Two different Bayesian classifiers, tree-augmented naive Bayes and Markov blanket estimating networks, were evaluated and compared by the authors (Elsayad 2010) [28] to build an ensemble model for the prediction of the severity of breast masses.

The proposed methodology is developed to help doctors decide whether or not to conduct a breast biopsy on a suspicious lesion after reviewing the findings of a mammogram. Based on Bayesian classifiers, they have been shown by the authors to be a competitive alternative to other approaches with medical applications. In the realm of emergency medicine, where BN is a beneficial approach because of its potent symbol and management of ambiguity, and where several alternatives are feasible depending on the data that has been provided, the authors (Krizmaric and Mertik 2008) [29] have decided to adopt Bayesian networks (BN). The symbolic representation inside Bayesian networks is what makes them such a powerful approach. The random forest (RF) classifier is an ensemble method that employs many different classification techniques. Every one of them may be put into action using a decision tree. Improved classification accuracy is achieved via the use of numerous decision trees (Dai, Chen, et al., 2018) [22]. In simple terms, the RF is an ensemble classifier made up of many decision trees that work together to improve efficiency and prediction accuracy. In Nguyen, Wang, et al., (2013) [30], researchers created an RF-based classifier. The algorithm they used has been trained on two datasets, and the findings are encouraging: it achieved high accuracy and good performance. Three classifiers—nearest neighbor (NB), radial basis function (RF), and k-nearest neighbors (KNN)—have been compared using the WDBC. The accuracy of these classifiers in predicting breast cancer tumors is evaluated by training and testing on the aforementioned dataset. The authors of this study found that although all of the classifiers they tested achieved detection accuracy rates of over 94%, KNN fared the best. Its accuracy is better than that of both the NB and RF classifiers. In addition to its superior accuracy, the KNN classifier also has a higher precision and F1-score (Sharma, Aggarwal, et al., 2018) [31]. According to Price and Lindquist, the ANN classifier outperforms the SVM, NB, or decision tree classifiers when using feature selection techniques. Its efficiency saw a 51 percent boost as a result of these changes (Kabiraj, Raihan et al., 2020) [32]. In Al-Azzam and Shatnawi (2021) [33], the authors evaluate two machine learning classifier models, extreme gradient boost (XGBoost) and RF, using a small dataset consisting of 275 examples. Using a limited dataset may reflect incorrect results; hence, the authors argue that a large dataset is necessary to validate their conclusions, even if their results suggest that RF has outperformed XGBoost when it comes to accuracy in detecting breast cancer. LR, Gaussian naive Bayes, RBF SVM, linear SVM, DT, RF, XGBoost, KNN, and gradient boosting are only some of the nine classification models that were examined in recent research. The Wisconsin Diagnosis Cancer Dataset is used for both training and testing the models. Based on the data, we can conclude that KNN is the most effective method for supervised learning, while LR is the most effective method for semi-supervised learning (Wang, Wang et al., 2020) [34]. One of the most incomplete methods to provide a trade-off between variance and bias is the ensemble learning technique. Classification performance may be improved by merging individual classifiers to construct an aggregated classification model, as has been shown in several studies. Stacking, boosting, and bagging are the three foundational methods of ensemble classification. The stacking method involves combining the results of several categorization models into a single one (Tang, Cai, et al., 2021) [35].

## 3. Proposed Methodology

In this paper, we applied six different categorization models for the diagnosis of breast cancer, including the random forest (RF), decision tree (DT), k-nearest neighbors (KNN), logistic regression (LR), support vector classifier (SVC), and linear support vector classifier (linear SVC). It is important to remember that the chosen dataset and objectives may dictate different hyperparameter values and other implementation-specific choices. Mammography, computed tomography, magnetic resonance imaging, positron emission tomography, blood testing, and genetic analysis all have their limitations when it comes to early diagnosis and prognosis. State-of-the-art techniques may require invasive treatments, incorrect diagnoses, and specialist knowledge. Early diagnosis, improved accuracy, personalized therapy, risk assessment, data integration, predictive prognosis, and drug development are just some of the ways machine learning technologies might boost cancer detection and prognosis. The existing process for identifying possible chemicals and treatments for cancer therapy is time-consuming and prone to mistakes; these strategies assist in speeding up the process.

Mammography is an X-ray imaging technique designed for breast tissue using differential X-ray absorption. It is primarily used for breast cancer screening, early detection, and diagnosis, detecting small abnormalities, e.g., tumors and microcalcifications. Ultrasound, on the other hand, uses high-frequency sound waves to create images of breast tissue. It is often used to complement mammography, distinguishing between solid masses and fluid-filled cysts and providing additional information about a breast abnormality’s characteristics. Ultrasound is also used to guide breast biopsies.

Thermography, also known as thermal imaging, captures heat patterns emitted by the body’s surface. It uses a specialized camera to measure the temperature of the skin’s surface, revealing temperature variations in areas with increased blood flow, such as around tumors. Thermography has been explored as a non-invasive tool for breast cancer screening but is not widely used as a standalone method due to concerns about accuracy and variability in interpreting results. It may be used as an adjunctive tool in certain cases or for breast health monitoring.

Mammography is the gold standard for breast cancer screening and is widely recommended in many countries. Ultrasound is often used as a complementary tool, particularly for dense breast tissue. Thermography is not recommended as a primary screening tool due to concerns about sensitivity and specificity. The choice of screening modality depends on a patient’s age, risk factors, breast density, and clinical circumstances and is determined by healthcare providers in consultation with patients.

Different ensemble approaches are used by the RF classifier and the KNN classifier, both of which are ensemble classifiers. The bagging method is crucial to RF. Tree-based classifiers make up this set. The stack classifier is a stacking-based classifier that uses the results of other classification models as inputs. By switching between classifiers that are predicated on various methods and use cases, we hope to improve the reliability of our findings.

**Figure 1:**
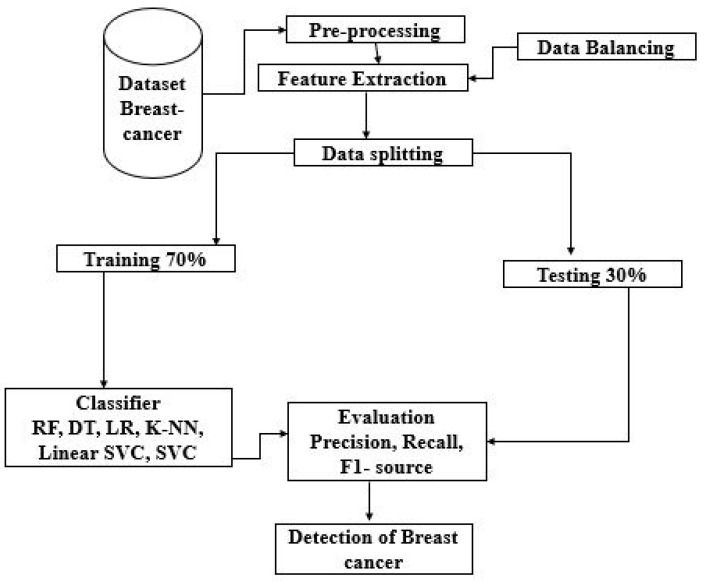
Proposed methodology.

Figure 1 shows our proposed methodology. In this figure, the data is taken from the dataset. Prepare the data to use the breast cancer dataset by loading it. Separate the information into features (X) and labels (y). For quantitative analysis of features, ensure each feature has the same effect on the algorithms by normalizing or standardizing them. Separate the data into a training set and a test set. Seventy percent for instruction and thirty percent for testing is an example of a frequent breakdown. For the evaluation and training of models, random forest (RF), decision tree (DT), k-nearest neighbors (KNN), logistic regression (LR) and linear support vector classifier (linear SVC) are used and the effectiveness of each algorithm is evaluated using the criteria provided. Select the most effective algorithm for spotting breast cancer. To determine which attributes are most important for algorithms such as RF and DT, perform a feature importance analysis. To better understand the algorithm’s decision-making process, decision trees may be represented graphically.

The objective of preprocessing is to replace duplication values in data. The data balancing is performed by using a data balancer. Then we performed feature extraction on it. The achieved data has been sent to the data splitting. Here, a specific algorithm is applied and hence the data is divided into two sets. One is training and the other is testing data. Our data were split into 70% training data and 30% testing data, and different ML classifiers were applied, such as DT, RF, LR, SVC, and KNN to find their individual detection and prevention accuracy levels for breast cancer. The raw breast cancer data is processed to resize the features with the Standard Scaler module as part of this research. Many estimators in the field of machine learning need the standardization of datasets. Typically, feature selection is used before any real learning is performed as a kind of preprocessing. However, without informative and discriminative characteristics, no algorithm can produce accurate predictions; hence, it is important to retain the most important features while decreasing the dataset size. We have used Python’s sci-kit-learn package to construct the feature-selection module. In addition, we have used too many criteria used by all selection procedures, making it impossible to prioritize the most important ones.

Implementations of the IMPEL algorithm are used for picture categorization. Collect a series of photos that will be used in the categorization process. Each picture in this collection should have a label indicating which category it belongs to. Creating variants of the photos by rotation, flipping, or adjusting brightness and contrast are all examples of preprocessing that may be used to prepare the images for analysis. It may be necessary to extract characteristics from photos to use them with common machine-learning techniques. Histogram of Oriented Gradients (HOG), Scale-Invariant Feature Transform (SIFT), and deep feature extraction using pre-trained convolutional neural networks (CNNs) such as VGG, ResNet, and Inception are used. For picture categorization, choose a machine-learning model that works well. Common alternatives are deep learning architectures: CNNs automatically learn useful characteristics from the input, making them very effective for image applications. If feature extraction is achieved, models such as support vector machines (SVMs), random forests, k-nearest neighbors (KNN), or logistic regression are used. For model instruction: evaluate the chosen model using the training data. Feed the photos into the CNN and tweak the model’s weights until the classification error is as small as possible. Use the validation dataset to fine-tune the model’s hyperparameters (such as the learning rate, batch size, and number of layers) for optimal performance.

Our study relied on three distinct modules for feature selection: the removal of low-variance features, univariate feature selection, and recursive feature elimination. Compared to using only one model, the prediction performance of ensembles of machine learning algorithms is often superior, as was proven by a breast cancer diagnostic model that won a machine learning competition. The information that is given here is classified using the following multimodal sets of machine learning algorithms: linear SVC, SVC, KNN, DT, RF, LR, DT, and logistic regression. There are now training, validating, and testing datasets available from the original dataset. The distribution is divided as follows: 30% to 70%. (1) Accuracy (2), precision (3), recall (4), and F1-score; all standard metrics have been used to evaluate the proposed model’s efficacy.

Testing the suggested model(s) created is part of evaluating an ML algorithm’s performance. In this study, the assessment is carried out by contrasting model findings with actual data values. In this step, known as the prediction phase, the performance of the models in identifying benign and malignant tumors is evaluated using the test dataset.

Malignant tumor classification is a major hurdle in the way of its discovery. They suggest machine learning (with SVMs) to categorize these tumors, using the Breast Cancer Wisconsin (diagnostic) dataset. The objective of this dataset is as follows:

- Learn the dataset and clean it up (if necessary).
- Create models for classifying cancers as either malignant or benign.
- Adjust the hyperparameters and evaluate the metrics of different classifiers.

A form of evolutionary algorithm (EA) known as genetic programming (GP) generalizes the genetic algorithm. GP is a model for testing and choosing the best option from a group of outcomes. GP creates a solution based on biological evolution and its basic process (mutation, crossover, and selection).

The usage of GP accounts for its adaptability; it can model systems even when the required models’ structure and salient characteristics are unknown. For the classification challenge in this study, GP enabled the system to search for models from a variety of potential model architectures while optimizing the pipelines shown as tree topologies. Based on the aforementioned primitives, such as the features selection decomposition, GP first constructs a certain number of pipelines. In other words, the order of operators changes as machine learning pipelines are reviewed to generate the highest possible categorization accuracy. A new generation is developed using the best prior pipelines after evaluating the present machine learning pipelines. Every pipeline is regarded as a unique member of GP. The three main operators make up the GP:

- Altering hyperparameters or adding or deleting a simple preprocessing step, such as Standard Scaler or the number of trees in a random forest, are examples of mutation operators.
- Crossover operator: using a 1-point crossover chosen at random, the crossover operator estimates that 5% of people will cross paths with one another.
- The major objective of the selection operator is to choose the top 20 people and create clones of them. The crossover or mutation operator may be used to communicate information between population members.

Table 2 presents the metrics description of the confusion matrix. In this table, we compare the anticipated outcomes with the actual numbers. The performance of the classifier is calculated using the matrix. Several performance metrics, including accuracy, an area under the precision, recall, sensitivity, and f1-score, may be used to assess the effectiveness of the ML model.

## 4. Results and Discussion

In this research, we have provided a standardized method for comparing the state-of-the-art using the assessment measures EDA dataset. At first, we collected the source from kaggle.com (accessed on 15April 2023). We have used Python Jupiter Notebook 6.4.12 for the simulation. Table 3 presents the first result of explanatory data analyses using different models. EDA resources include Python extensions such as Pandas, Seaborn, Plotly, and Bokeh. Pandas is a robust library for data analysis, offering DataFrames and Series. Seaborn is a high-level interface for creating statistical visualizations, while Plotly is a flexible framework for creating interactive visualizations such as dashboards and 3D plots. Bokeh is a library for creating interactive dashboards for online use. In this graph, the predictions are used with the subsets of the dataset. Each set consists of 70% of the training data and 30% of the test data. The table summarized and compared with id, radius_mean and other measures. 

Keep in mind that the variety of the individual models and their capacity to capture various features of the data are essential to the success of an ensemble model. Throughout the process, careful model selection, training, and assessment are crucial. The ensemble model must be monitored and maintained when new data becomes available or the clinical setting changes.

The above data analysis method would be incomplete without the exploratory data analysis (EDA) phase. Analyzing data entails visualizing it to draw conclusions, recognize trends, spot outliers, and make hypotheses. EDA is useful for gaining a grasp of the dataset’s structure and properties before moving on to more complex statistical and machine learning methods. Understanding the features of the dataset, spotting data quality concerns, and identifying early patterns that might drive further analysis and modeling choices are all accomplished using EDA, making it a crucial phase in the data analysis process. It is also vital to the trustworthiness of the results obtained from the data.

**Figure 2:**
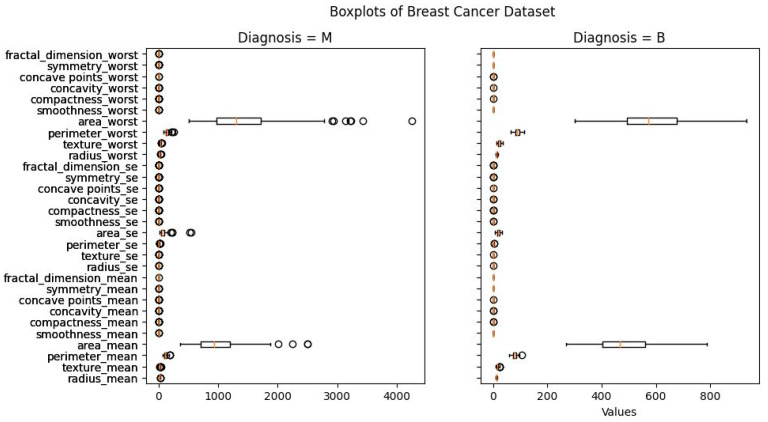
Boxplots of breast cancer dataset.

Figure 2 presents the boxplots of the breast cancer dataset. In this figure, these boxplots present the distribution of breast cancer detection scores. There is an anomaly in all the graphs we acquired. If we remove them, the data’s median will go down, and that might provide a challenge in accurately identifying cancers, particularly if a malignant tumor is misidentified as benign. We will only remove anomalies in benign tumors, however, so as not to interfere with the detection of dangerous cancers. After processing, the maximum value of the area ‘worst’ is 932.7, down from 1210.0 before processing. The maximum area of mean value has similarly decreased, from 992.1 before processing to 788.5. This might lead to false positives, in which benign tumors are mistakenly labeled as cancerous. On the other hand, this strategy is preferable to the alternative. This diagnosis has the potential to save lives by detecting cancers at an early stage when treatment is most effective.

Breast cancer screening involves mammography to detect the early stages of the disease, identifying abnormalities such as tumors or microcalcifications. The procedure involves gently compressing the breast between two plates, resulting in two X-ray images. Radiologists review the images to identify suspicious findings, such as masses, abnormal densities, microcalcifications, or architectural distortions. Mammography results are often reported using the Breast Imaging Reporting and Data System (BI-RADS), which categorizes findings into levels. If a mammogram is assigned BI-RADS 0, further evaluation, such as additional imaging or a biopsy, is needed. Follow-up tests may be recommended to confirm or rule out cancer, such as ultrasound, MRI, or a breast biopsy, depending on the results and BI-RADS category.

When a mammographer examines a patient’s breast, she or he will put the breast on the mammography machine’s plate and gently compress it with a second plate to spread out the breast tissue so that the image may be taken. Each breast is X-rayed twice, once from the top down (cranio-caudal view) and once from the side (mediolateral oblique view), as is common protocol. If more views are required, that option is available.

**Figure 3:**
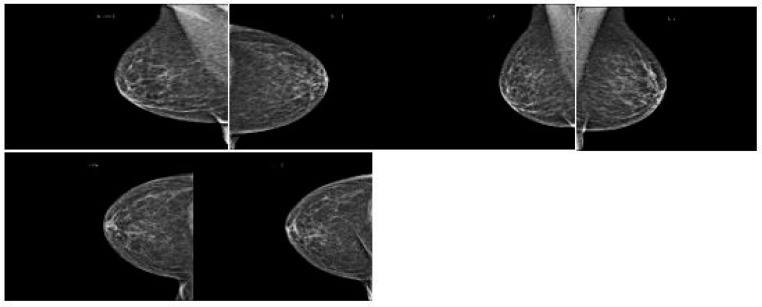
Breast cancer mammography screening.

**Figure 4:**
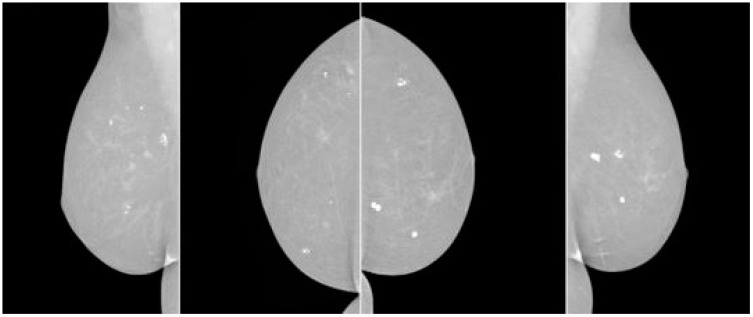
Breast cancer look on mammography.

Figure 3 shows how breast cancer on mammograms can vary depending on stage, size, and location. Radiologists examine for abnormalities and patterns to detect breast cancer. Common findings include masses, microcalcifications, architectural distortions, asymmetries, spiculated borders, and nodules. Masses, microcalcifications, and architectural distortions are early signs of breast cancer, while microcalcifications are tiny calcium deposits in breast tissue. Figure 4 show the architectural distortions. These may appear as irregularities in the breast’s structure, while asymmetries indicate differences in appearance between the left and right breasts. Speciated borders, jagged or spiky edges, and nodules are small rounded masses that are assessed for potential malignancy.

**Figure 5:**
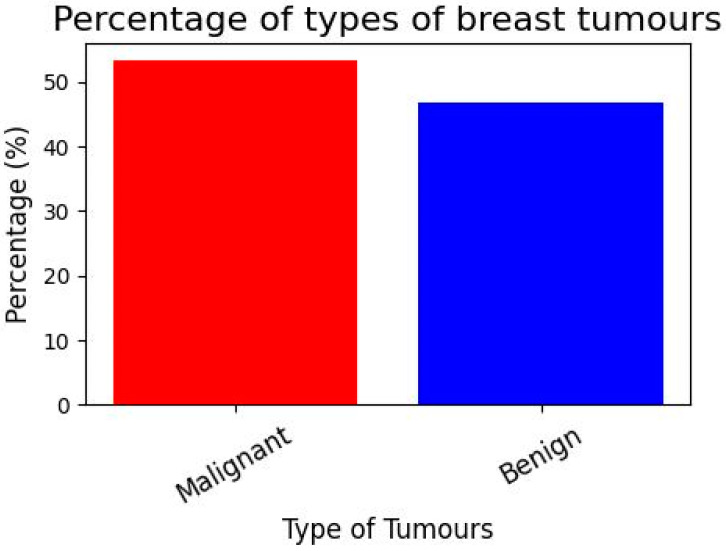
Breast cancer type percentages.

Figure 5 demonstrates the various types of breast cancer percentages. We have a dataset with the following variables, cancer type (malignant or benign) and group of people (young adults, middle-aged adults, and elders). Make a graph showing the distribution of instances by age group, and whether they were malignant or not. We used a breast cancer dataset including age- and diagnosis-related categories. Pandas are used to determine the proportion of instances in each age group for both malignant and benign diagnoses. Finally, to prepare the data for the graph, a stacked bar graph is drawn to show the distribution of cases by age and diagnosis. Age, gender, geography, and demographics are only a few of the variables that might affect the breast cancer prevalence rate. Other influences include one’s way of life, one’s genetic makeup, and one’s level of access to medical treatment. According to the WHO, breast cancer is the most frequent cancer among women worldwide. Case rates of breast cancer differ significantly across geographic locations. The proportion is often greater in affluent nations due to widespread screening programs and public education efforts than in underdeveloped countries due to scarcer resources and less awareness. For accurate and up-to-date data on breast cancer, the American Cancer Society (ACS), the National Cancer Institute (NCI), the World Health Organization (WHO), or other respected organizations are excellent resources.

A dataset is partitioned into K equal-sized subgroups as part of the breast cancer detection technique known as K-fold cross-validation. One of the K subsets is used as the test set, while the other K-1 subsets are used for training. The model is trained and assessed K times. To evaluate the model’s generalization to new data, performance measures are computed on the test set. The performance indicators from each fold are averaged after all K iterations to obtain a thorough analysis. Robustness, overfitting detection, optimum hyperparameter tuning, bias and variance evaluation, and realistic performance estimates all depend on K-fold cross-validation. It ensures that the assessment is more reliable and less reliant on a certain data split, enabling more precise and accurate forecasts of the model’s performance on new unseen data.

Breast cancer detection using machine learning: K-fold cross-validation ensures that the chosen model’s performance is thoroughly assessed, helping one to build a reliable and robust diagnostic tool. It is an essential step in the model development process to ensure that the chosen system can provide accurate and consistent results on a variety of patient data.

**Figure 6:**
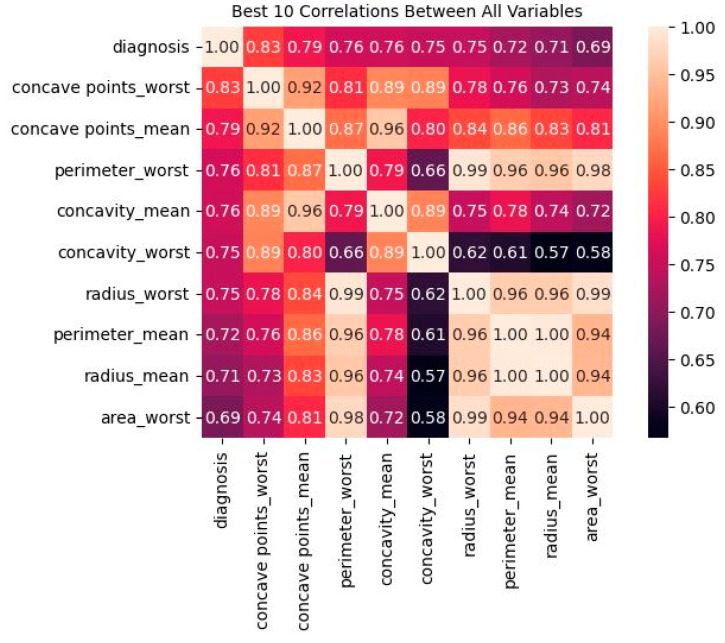
Correlation between the ten best variables.

Figure 6 presents the best-ten correlation results between all variables. The values on the *y*-axis include diagnosis, radius_mean testure_mean, perimeter_mean, area_mean, smoothness_mean, etc. A correlation coefficient between −1 and 1 indicates no association at all, whereas a positive or negative number indicates a strong relationship between the two variables. It is important to note that the linear connection between variables is all that can be measured using correlation. There is a link of 69% or higher between each of these factors and the outlook for the patient.

**Figure 7:**
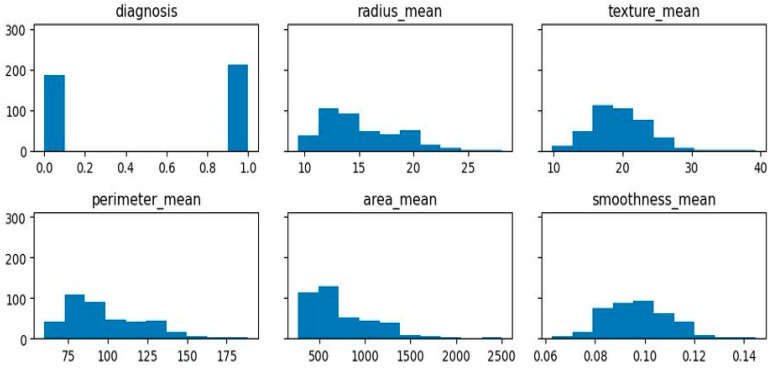
Calculated correlations by absolute value.

Figure 7 presents the calculated correlations of diagnosis, radius_mean, texture_mean, etc. by absolute value. The diagnostic relationship between features will be studied to test this idea. The vast majority of data is right-skewed, meaning that its right-hand tail is much longer or fatter than its left-hand tail. This indicates that the mean is higher than the median and that there are more observations of high values than low ones. If the breast cancer dataset has a right-skewed distribution of characteristics, then the majority of patients will have smaller values for certain aspects, while a small percentage will have larger values. This may suggest that the importance of some characteristics varies with the kind of tumor being studied.

**Figure 8:**
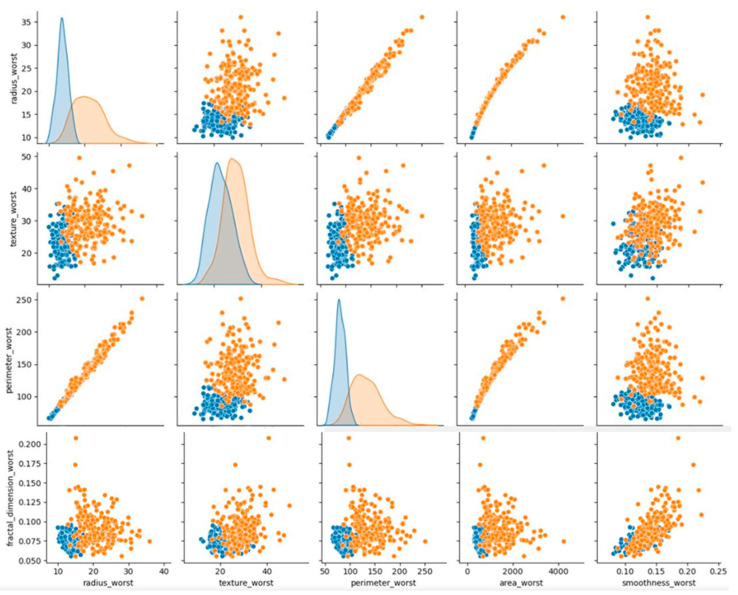
Breast cancer diagnosis graph.

Figure 8 presents the breast cancer diagnosis. In this figure, the color area indicates the most that the most patients have lower values in some features and only a few have very high values. The majority of the traits are strong concerning each other, as demonstrated in the graphs. Additionally, compared to benign tumors, malignant tumors have larger values for the same characteristics. Additionally, malignant tumor outliers exhibit higher variation than benign tumor outliers and are better represented in clusters after they have not been treated.

Most of the characteristics are rather robust in comparison to the other traits, as illustrated by the graphs. For the same characteristic, malignant tumors tend to have higher values. In addition, after the extreme cases have been removed, data from malignant tumors show higher variation than data for benign tumors. Most of the characteristics are rather robust in comparison to the other traits, as illustrated. When comparing benign and malignant tumors, the malignant tumor always has greater values for the same trait. Additionally, data from malignant tumors, which are better represented in clusters due to the absence of outliers, show higher variation.

**Figure 9:**
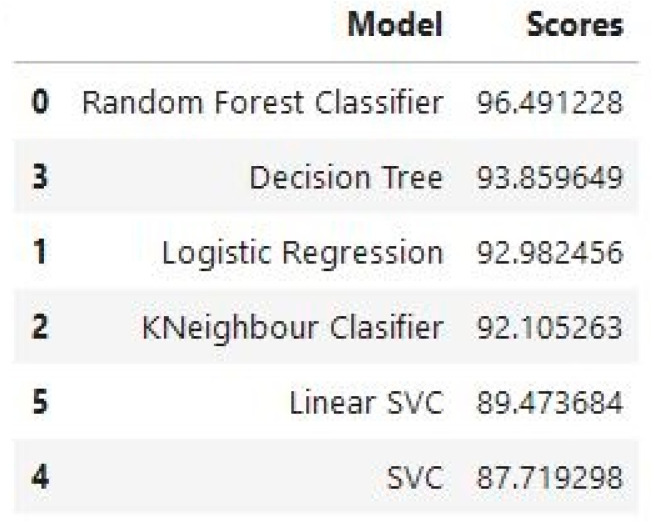
Classifiers accuracy result.

Figure 9 shows 0 to 100%, and our highest accuracy is 96.49% by random forest. ML has the potential to greatly improve the identification and diagnosis of breast cancer. However, to successfully integrate technology into clinical practice, there are technological, moral, and legal obstacles to overcome. The objective is to develop a more precise, approachable, and patient-centric approach to breast cancer diagnosis and care as research progresses and technology advances.

Figure 9 shows the accurate results of all classifiers that we used. This figure shows the random forest as the best result which achieved 96.49% accuracy. The description of others is given below.

- Random forest models have the highest accuracy values, indicating better performance in predicting the target variable compared to other models.
- Decision tree and KNN have higher values than LG and SVC and are comparable to other models, indicating that they may not be the best models for predicting the target variable.
- Logistic regression and SVC have similar values, indicating they have comparable performance in predicting the target variable.

When it comes to detecting cancer, random forest may be a viable alternative because of its versatility, ease of interpretation, and ability to determine which traits are most crucial to the categorization decision-making process.

**Figure 10:**
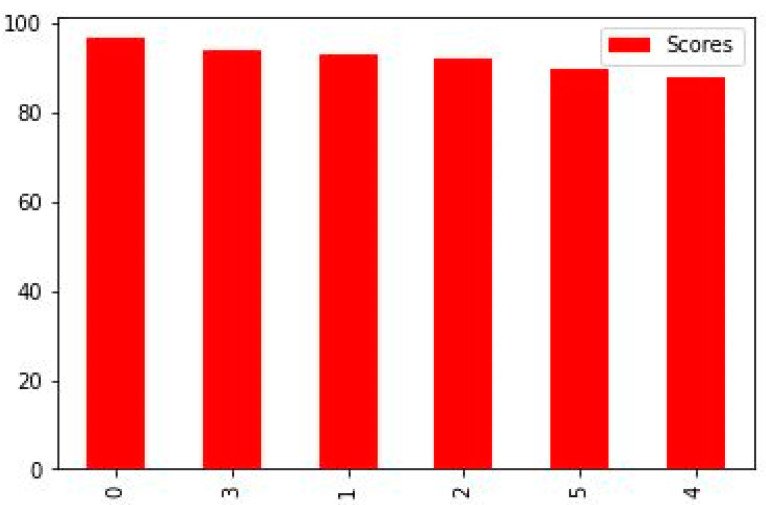
Accuracy graph.

Figure 10 shows the graphical accuracy result of all classifiers. In this graph, we used RF, DT, KNN SVC, LR, and linear SVC.

The selection of an algorithm for breast cancer diagnosis in the real world should consider the unique needs of the clinical setting as well as the available computational resources. The best method should be chosen based on factors including the size of the dataset, hardware capabilities, and whether real-time or batch processing is required. The computational efficiency of selected algorithms may also be increased via the use of parallelization and optimization methods, ensuring their viability and efficacy for clinical applications.

**Figure 11:**
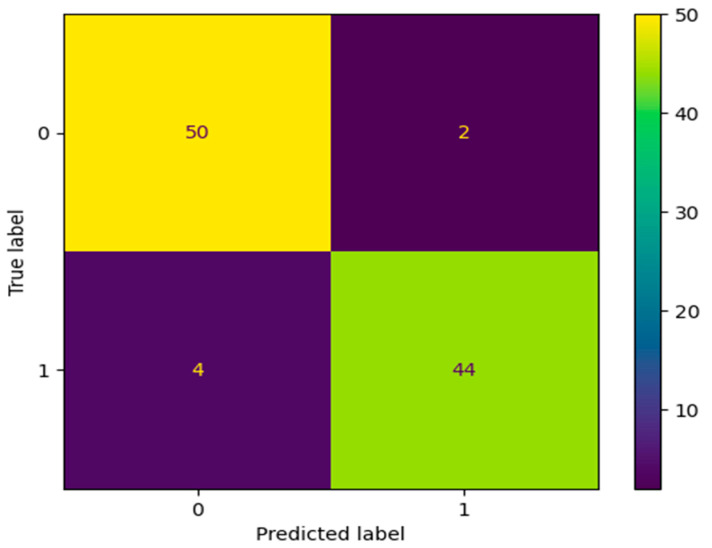
Predicted value.

Figure 11 shows the predicted value of TP, FP, FN, and TN. In this confusion matrix, each entry represents the number of occurrences that share a certain set of labels for both the actual and expected classes. This confusion matrix, in particular, contains:

- Fifty (50) instances (True Positives)—malignant tumor classifications
- Two (2) instances (False Positives)—being tumors classified as malignant tumors
- Four (4) instances (False Negatives)—malignant tumors classified as benign tumors
- Forty-four (44) instances (True Negatives)—being tumors classifications

A large number of true positives and true negatives in the confusion matrix indicates that the model has successfully predicted both groups. The model’s success may be seen in its low rate of false positives and false negatives.

## 5. Conclusions and Future Work

This study examines six different categorization models for breast cancer classification using the Breast Cancer Wisconsin (diagnostic) dataset. The data is processed using the Standard Scaler module and feature selection is performed using Python’s scikit-learn package. The models were developed using multimodal sets of machine learning algorithms, including linear SVC, SVC, KNN, DT, RF, LR, DT, and logistic regression. The study used a confusion matrix to compare anticipated outcomes with actual numbers and assessed performance metrics such as accuracy, area under the precision, recall, sensitivity, and f1-score. The results were summarized and compared using exploratory data analysis. The study found that maximum area worst and maximum area_mean values decreased after processing, potentially leading to false positives. The correlation between variables in breast cancer diagnosis is crucial for understanding the relationship between features and patient outlook. Random forest models have the highest accuracy values, followed by decision tree and KNN. Logistic regression and SVC have similar performance in predicting target variables. Random forest may be a viable alternative for detecting cancer due to its versatility, ease of interpretation, and ability to identify crucial traits for categorization decision-making. Breast cancer is a prevalent disease affecting women worldwide, with machine-learning approaches potentially impacting early detection and prognosis. The disease is classified into two subtypes: invasive ductal carcinoma (IDC) and ductal carcinoma in situ (DCIS). Early detection is crucial for successful treatment, and appropriate screening technologies are essential. Mammography, ultrasonography, and thermography are common imaging modalities for detecting breast cancer. Advancements in artificial intelligence have made mammography more accurate, and deep learning models are being developed to recognize breast cancer in computerized mammograms. Breast MRI is a sensitive imaging technique with excellent sensitivity and specificity, and convolutional neural networks and AI are emerging in healthcare to improve image processing and reduce human eye recognition.

Future research on breast cancer diagnosis using ML might explore these and other possibilities. To make substantial strides forward in the detection and treatment of breast cancer, continued research and cooperation between data scientists, medical experts, and researchers is essential.

## Figures and Tables

**Table 1:** Existing diagnostic method, advantages, and limitations.

Existing Diagnostic Method	Advantages	Limitations
Mammography	Well-established	Limited sensitivity in dense breast tissue
	Widely accessible	False positives/negatives
	Detects structural changes and calcifications	False positives/negatives
Ultrasound	No radiation	Limited specificity
	Useful for dense breasts	Operator-dependent
	Differentiates cysts from solid masses	Limited detection in deep tissues
MRI (Magnetic Resonance Imaging)	High sensitivity	High cost
	No radiation	Longer exam duration
	Detailed soft tissue visualization	Requires specialized expertise to detect benign lesions
Biopsy (Fine Needle Aspiration or Core Needle Biopsy)	Provides tissue samples for definitive diagnosis	Invasive and uncomfortable
	High diagnostic accuracy	Small risk of complications
		Requires skilled medical staff
		Sample may not be representative
Clinical Breast Examination (CBE)	No radiation	Limited sensitivity
	Low cost	Dependent on examiner’s expertise
	Can detect palpable masses	May miss non-palpable masses
Genetic Testing (BRCA1/BRCA2 Testing)	Identifies genetic mutations linked to increased risk	Applicable to specific subsets of patients
	Enables targeted prevention and treatment strategies	Limited to hereditary breast cancer cases

**Table 2:** Description of metrics.

Metrics	Description		
Confusion Metrics		Positive	Negative
	Positive	True Positive (TP)	False Positive (FP)
	Negative	False Negative (TP)	True Negative (TN)
		Precision $=\frac{TP}{TP+FP}$	

**Table 3:** Exploratory data analysis.

(**a**)										
**id**	**Diagnosis**	**Radius_Mean**	**Texture_Mean**	**Perimeter_Mean**	**Area_Mean**	**Smoothness_Mean**	**Compactness_Mean**	**Concavity_Mean**	**Concave Points_Mean**	**Radius_Worst**
**0**	842302 M	17.99	10.38	122.80	1001.0	0.11840	0.27760	0.3001	0.14710	25.38
**1**	842517 M	20.57	17.77	132.90	1326.0	0.08474	0.07864	0.0869	0.07017	24.99
**2**	84300903 M	19.69	21.25	130.00	1203.0	0.10960	0.15990	0.1974	0.12790	23.57
**3**	84348301 M	11.42	20.38	77.58	386.1	0.14250	0.28390	0.2414	0.10520	14.91
**4**	84358402 M	20.29	14.34	135.10	1297.0	0.10030	0.13280	0.1980	0.10430	22.54
(**b**)										
**Texture_Worst**	**Perimeter_Worst**	**Area_Worst**	**Smoothness_Worst**	**Compactness_Worst**	**Concavity_Worst**	**Concave Points_Worst**	**Symmetry_Worst**	**Fractal_Dimension_Worst**	**Unnamed: 32**	
17.33	184.60	2019.0	0.1622	0.6656	0.7119	0.2654	0.4601	0.11890	NaN	
23.41	158.80	1956.0	0.1238	0.1866	0.2416	0.1860	0.2750	0.08902	NaN	
25.53	152.50	1709.0	0.1444	0.4245	0.4504	0.2430	0.3613	0.08758	NaN	
26.50	98.87	567.7	0.2098	0.8663	0.6869	0.2575	0.6638	0.17300	NaN	
16.67	152.20	1575.0	0.1374	0.2050	0.4000	0.1625	0.2364	0.07678	NaN	

## Data Availability

Not applicable.
